# Screening tests for distal symmetrical polyneuropathy in Latin American patients with type 2 diabetes mellitus

**DOI:** 10.1590/2359-3997000000283

**Published:** 2017-06-23

**Authors:** Nicolás Gómez-Banoy, Virginia Cuevas, Fernando Soler, Maria Fernanda Pineda, Ismena Mockus

**Affiliations:** 1 Laboratorio de Lípidos y Diabetes Facultad de Medicina Universidad Nacional de Colombia Bogotá Colombia Laboratorio de Lípidos y Diabetes, Facultad de Medicina, Universidad Nacional de Colombia, Bogotá, Colombia

**Keywords:** Type 2 diabetes, diabetic neuropathy, complications, diagnostic test

## Abstract

**Objective:**

This cross sectional study intended to evaluate two bedside tests (Neuropad and VibraTip) as screening tools for distal symmetrical polyneuropathy (DSPN) in Latin American patients with type 2 diabetes mellitus (T2D).

**Subjects and methods:**

Ninety-three Colombian patients diagnosed with T2D were recruited. Anthropometric variables, glycemic control parameters, lipid profile and renal function were assessed for each patient. DSPN was defined by a Michigan Neuropathy Screening Instrument (MNSI) clinical score greater than 2. Both Neuropad and Vibratip tests were applied to each patient. Contingency analyses were performed to evaluate the diagnostic power of both tools.

**Results:**

The prevalence of DSPN determined clinically by MNSI was 25.8%. DSPN in these patients was associated with age, worsening renal function, and insulin treatment. The sensitivity and specificity of the Neuropad test for DSPN was 66.6% and 63% respectively. Its negative predictive value (NPV) was 84.6%. The VibraTip test exhibited a sensitivity of 54.1% and specificity of 91.3%, with a NPV of 85.1%.

**Conclusion:**

Neuropad and VibraTip are reliable screening tools for DSPN in Latin American population. VibraTip presents a considerable diagnostic power for DSPN in this population. Further studies regarding the cost-effectiveness of these tools in clinical practice are needed.

## INTRODUCTION

Diabetic neuropathy (DN) is a common micro-vascular complication of type 2 diabetes mellitus (T2D). It is estimated that DN affects 26-47% of diabetes patients in the United States, and it comprises a wide variety of clinical syndromes (1). Specifically, distal symmetrical polyneuropathy (DSPN) is the most common presentation of DN, and it accounts for 50% of neuropathies associated to diabetes (2). In Latin America, the prevalence of DN has been reported around 55% in some countries (3), and in Colombia it is present in 68% of hospitalized T2D patients (4).

The diagnosis of DSPN is primarily clinical, involving a detailed medical history and neurological examination. The Toronto Consensus Panel on Diabetic Neuropathy defined specific diagnostic criteria based on various signs and symptoms (5), however standardized tests and questionnaires have been developed and are frequently used in clinical practice. One of this is the Michigan Neuropathy Screening Instrument (MNSI), which consists of a self-administered questionnaire and a 5-item physical examination. This tool has been widely validated (6,7) and used to determine the presence of peripheral diabetic neuropathy in various longitudinal studies (8).

In order to improve the diagnosis and screening of peripheral neuropathy in T2D patients, new tests have been developed in the last years (9). Among these, Neuropad and VibraTip are characterized by being bedside, simple and accessible tools to evaluate the presence of small fiber and large fiber dysfunction respectively. Neuropad is a test designed to measure sudomotor dysfunction in the foot through a cobalt II salt-impregnated patch applied to the soles’ skin. The reaction from the water produced in the sweat glands and the mentioned chemical will gradually change the color of the patch from blue to pink. The Neuropad has been evaluated extensively in European T2D patients as a reliable screening test for DSPN (10). The VibraTip is a handheld device designed to test vibration perception by producing stimulus of 128 Hz; it has been validated as a useful test for diabetic neuropathy screening in European patients with T2D (11). Due to the potential usefulness of both Neuropad and VibraTip in outpatient clinical practice, and the limited data regarding the validity of these tests in Latin American patients, we sought to evaluate the diagnostic performance of Neuropad and VibraTip for DSPN in Colombian patients with T2D.

## SUBJECTS AND METHODS

### Patients

The patients considered for this study were outpatients belonging to the “Program for the Prevention of Diabetes Complications” of the Lipids and Diabetes Laboratory, Faculty of Medicine, National University of Colombia. The study was conducted in the premises of the National University of Colombia, once approved by the institutional Ethics Committee in accordance with the Helsinki Declaration. All patients were diagnosed with T2D based on the American Diabetes Association (ADA) criteria (12). Exclusion criteria included diagnosed or established neuropathy from other etiology (according to clinical history), active neoplastic or autoimmune disease, acute exacerbation of chronic disease, pregnancy, and age under 18.

### Physical examination and general tests

During a period of two months, each patient received their usual follow-up medical exam, during which anthropometric variables, body mass index (BMI) (kg/m^2^) and waist circumference (cm) were determined. Blood pressure (mmHg) was determined clinically with a mercury sphygmomanometer. Results from their routine metabolic and lipid profile were assessed: plasma glucose (mg/dL), triglycerides (mg/dL), total cholesterol (mg/dL), HDL cholesterol (mg/dL), and serum creatinine (mg/dL). LDL cholesterol was calculated using the Friedewald formula (13). Haemoglobin A1c (HbA1c) was reported in NGSP units (%) and in IFCC units (mmol/mol). All laboratory exams were determined from blood samples taken after an 8 hour fast, in a range of 1 to 3 days previous to medical consultation. Estimated glomerular filtration rate (eGFR) was calculated using the Chronic Kidney Disease Epidemiology Collaboration (CKD-EPI) equation as recommended by current guidelines (14).

On the same day of medical examination, the objectives and procedures of the study were explained to each patient, and a signed informed consent form was obtained from all participants. Patients were then asked to answer the MNSI 15 – item symptom-based questionnaire. According to previous validation studies, if the summing of abnormal answers was greater than 7, the questionnaire was considered positive for DSPN (15). Afterwards, participants underwent neurological examination of both feet based on the MNSI clinical test, involving: 1) inspection to detect deformities, dry skin, calluses, infection, 2) inspection to detect ulcerations, 3) grading of ankle reflexes, 4) assessment of vibration perception at great toe, and 5) 10-g monofilament testing. Each foot was examined individually. Previous validation studies established a maximum score of 8 for the MNSI (15); however, they did not include the 10-g monofilament test. Here, we established a maximum possible score of 10, in accordance with recent epidemiological studies (8,16). In this analysis, the presence of DSPN was defined by a score greater than 2 (> 2) in the clinical examination component of MNSI (8).

### Neuropad and VibraTip tests

The Neuropad and VibraTip tests were performed by two qualified primary-care physicians blinded to results of the MNSI.

For Neuropad testing, patients were asked to remove their footwear and socks 10 minutes before applying a Neuropad patch to each of their soles, in a callus-free area between first and second metatarsal head. A test was considered positive for DSPN if the patch remained completely blue or had a patchy appearance 10 minutes after application in one or both soles.

For the VibraTip test, the physician touched the hallux of each foot twice with the device. In one of the touches, a vibratory stimulus would be applied. Then, the patient was asked in which of the two he/she felt a vibration. A test was considered positive for DSPN if the patient failed to detect the vibratory stimuli in one or both feet (11).

### Statistical analysis

Data are presented as means ± standard deviation (SD). Statistical Analyses were conducted using the Prism 6.0e software for Mac (GraphPad Software, Inc). Patients were divided into two groups for comparison: subjects with clinical DSPN (MNSI > 2) and subjects without DSPN (MNSI ≤ 2). A student’s T test was used to compare the means of important variables in both groups. To compare proportions between both groups, a Chi-square test was performed. Measures of diagnostic performance (sensitivity, specificity, negative and positive predictive values) were calculated as previously described (17). All tests were two-tailed. A p value of less than 0.05 was considered significant.

## RESULTS

### DSPN prevalence and clinical characteristics of the population

Of the 120 eligible patients belonging to the “Program for the Prevention of Diabetes Complications”, 93 matched with the inclusion and exclusion criteria and were included in the present study. Table 1 shows the demographic and clinical characteristics of the participants. Fifty-two men and 41 women participated. Mean age at the time of the study was 70.8 ± 7.7 years. The prevalence of neuropathy was 25.8% (24 out of 93 patients presented a MNSI clinical score > 2). Men presented with a prevalence of 30.7% whereas women had 19.5%, with no statistical difference between both groups (p = 0.218). Regarding the MNSI symptom-based questionnaire, only 4.3% of patients had a score greater than 7. The proportion of patients with evidence of CKD (eGFR < 60) was 21.5%. The duration of T2D in the overall population was 10 ± 8.2 years. Patients with DSPN presented slightly longer T2D duration than patients without DSPN, but the difference was not significant (p = 0.27).

**Table 1:** Clinical characteristics of total T2D patients and patients with and without neuropathy

	All patients (n = 93)	Patients with neuropathy (MNSI > 2)	Patients without neuropathy (MNSI ≤ 2)	p
Age (years)	70.8 ± 7.7	75.8 ± 7.3	69.1 ± 7	< 0.001
T2D duration (years)	10.0 ± 8.2	11.5 ± 7.5	9.4 ± 8.4	0.27
Hemoglobin A1C (%)	7.3 ± 1.4	7.5 ± 1.1	7.2 ± 1.5	0.376
Fasting plasma glucose (mg/dL)	130 ± 43	129 ± 35	130 ± 45	0.9241
Triglycerides (mg/dL)	149 ± 55	151 ± 50	148 ± 56	0.829
Total cholesterol (mg/dL)	172 ± 36	170 ± 27	173 ± 38	0.705
HDL cholesterol (mg/dL)	41.9 ± 7.2	40.8 ± 5.8	42.3 ± 7.6	0.392
LDL cholesterol (mg/dL)	100.5 ± 31.9	98.6 ± 21.5	101.1 ± 34.7	0.738
Creatinine (mg/dL)	1.0 ± 0.2	1.1 ± 0.3	0.9 ± 0.2	< 0.001
eGFR (ml/min/1.73 m^2^)	72 ± 14.5	63.1 ± 14.8	75.1 ± 13	< 0.001
Urinary albumin (mg/L)	54.9 ± 135.6	106.3 ± 225	37 ± 77	< 0.05
BMI (kg/m^2^)	27.8 ± 3.5	27.5 ± 3.4	27.9 ± 3.6	0.647
Subjects treated with metformin (%)	63,4%	54.2%	66.6%	0.273
Subjects treated with insulin (%)	29%	50%	21.7%	< 0.01
Subjects treated with sulphonylureas (%)	19.4%	16.6%	20.3%%	0.698
Subjects treated with DPP-4 inhibitor (%)	15.1%	12.5%	15.9%	0.684
Subjects treated with ACE inhibitor (%)	18.3%	16.6%	18.8%	0.316
Subjects treated with ARB (%)	46.2%	66.6%	39.1%	< 0.05
Subjects treated with statins (%)	64.5%	66.6%	63.7%	0.798

Data are expressed in Mean ± SD. P values depict differences between patients with and without neuropathy. T2D: type 2 diabetes; HDL: High Density Lipoprotein; LDL: Low Density Lipoprotein; eGFR: estimated Glomerular Filtration Rate; BMI: Body Mass Index; DPP-4: Dipeptidyl Peptidase 4; ACE: Angiotensin Converting Enzyme; ARB: Angiotensin Receptor Blocker.

Also, the presence of DSPN was associated with increasing age (p < 0.001), increased creatinine values (p < 0.001), lower eGFR (p < 0.001), and higher urinary albumin excretion (p < 0.05). In terms of medication usage, DSPN was associated with insulin treatment (p < 0.01), and usage of angiotensin receptor blockers (ARB) (p < 0.05).

### Diagnostic performance of Neuropad and VibraTip

A total of 41 patients presented an abnormal Neuropad test. In patients without clinical DSPN (MNSI ≤ 2) 36.2% presented abnormal Neuropad test, whereas in patients presenting clinical DSPN (MNSI > 2) the proportion was 66.6% (p < 0.05). Table 2 depicts the diagnostic utility of the Neuropad test for DSPN. The specificity and sensitivity are very similar, and it presents a high negative predictive value (NVP).

**Table 2:** Diagnostic performance of Neuropad for detection of diabetic neuropathy

Reference Test	Sensitivity	Specificity	PPV	NPV
5-item MNSI	66,6%	63.8%	39%	84.6%
128-Hz-tuning fork	39%	82.9%	64%	63.2%
10-g monofilament	24.3%	94.2%	76.9%	61.2%
Ankle reflex	60.9%	71.2%	62.5%	69.8%
VibraTip	29.2%	86.4%	63.1%	60.8%

PPV: positive predictive value; NPV: negative predictive value. MNSI: Michigan Neuropathy Screening Instrument.

Regarding the VibraTip test, a total of 19 patients presented an abnormal result. In participants without clinical neuropathy 8.7% had an abnormal VibraTip test, whereas the proportion in the neuropathy group was 54.2% (p < 0.05). Table 3 shows the diagnostic performance of VibraTip. Importantly, it has a high specificity value.

**Table 3:** Diagnostic performance of VibraTip for detection of diabetic neuropathy

Reference Test	Sensitivity	Specificity	PPV	NPV
5-item MNSI	54.2%%	91.3%	68.4%	85.1%
128-Hz-tuning fork	73.6%	85.1%	56%	91.7%
10-g monofilament	42.1%	93.2%	61.5%	86.2%
Ankle reflex	89.4%	68.9%	42.5%	96.2%
Neuropad	63.1%	60.8%	29.2%	86.5%

PPV: positive predictive value, NPV: negative predictive value. MNSI: Michigan Neuropathy Screening Instrument.

### Diagnostic performance of individual diagnostic tools

Table 4 shows the diagnostic power of each individual component of the MNSI. Interestingly, the 10-g monofilament shows the highest sensitivity, followed by the 128- Hz-tuning fork.

**Table 4:** Performance of all diagnostic tools for detection of diabetic neuropathy (MNSI > 2)

Reference Test	Sensitivity	Specificity	PPV	NVP
128-Hz-Tuning Fork	70.8%	88.4%	68%	89.7%
10-g monofilament	54.2%	100%	100%	86.3%
Ankle reflex	75%	68.1%	45%	88.7%
Neuropad	66.7%	63.8%	39%	84.6%
VibraTip	54.2%	91.3%	68.4%	85.1%

PPV: positive predictive value; NPV: negative predictive value. MNSI: Michigan Neuropathy Screening Instrument.

## DISCUSSION

In the present study we aimed to study the diagnostic performance of two new screening tools for diabetic DSPN, Neuropad and Vibratip. Our work demonstrates that both Neuropad and Vibratip are reliable tests for the screening of clinical DSPN measured by the MNSI. Additionally, Vibratip showed a better diagnostic performance than Neuropad.

The prevalence of diabetic neuropathy measured by the MNSI was 25.8% in our population, which is low compared to other studies in Colombian and Latino patients (3,4). A cross-sectional study in Mexico reported prevalence as high as 69% of DSPN using the 5-item clinical MNSI (18). Similar to our results, studies in European population have shown 28.8% of DSPN prevalence in T2D patients using the 5-item clinical MNSI (16), and 30.6% using a modified version of the clinical MNSI (19). Regarding the MNSI questionnaire, previous studies have shown that it underestimates the prevalence of diabetic neuropathy, with values bellow 5% (7,19), similar to our study (4.3%). For this reason, the clinical component of the MNSI was used to determine the presence of DSPN.

One of the potential reasons for the low neuropathy prevalence in our population is that these patients belong to a specialized screening program for diabetes complications and are carefully followed and treated to achieve adequate glycemic control (mean HbA1c of 7.3). Furthermore, there is a low incidence of other micro-vascular complications, such as diabetic nephropathy (21.5%). Consistently, the patients presenting DSPN were significantly older, showed worsening renal markers, and presented a more advanced metabolic disease (insulin treatment).

Neuropad is an adhesive patch utilized to determine skin hydration status in the soles of the foot; this way, it measures the degree of sudomotor dysfunction and, indirectly, small fiber functionality (20). This test has been widely validated in European countries, where it has been utilized as a screening test for DSPN due to its high sensitivity values (65.1%-100%), moderate specificity (32-78.5%), and high negative predictive values (63-100%) (9). A recent study evaluated the diagnostic performance of Neuropad in Latin American patients with T2D, finding a sensitivity of 77.8% and a NPV of 63.8% for DSPN measured by a sign-based scale (Michigan Neuropathy Disability Score). Interestingly, they found a better correlation between Neuropad and cardiovascular autonomic neuropathy (CAN) than with DSPN (21). Similarly, in our study we found sensitivity values (66.6%) comparable to those reported in the literature, and, importantly, Neuropad presented a high NPV (84.6%) for DSPN. Taken together, our findings confirm the utility of Neuropad as a reliable screening test for DSPN in Latin American population. One of the potential advantages of this tool is that its results may be witnessed and understood by patients; this may aid the physician in promoting self-consciousness and adequate foot care habits. This is especially important, since patient education alone does not seem to lead to clinically relevant reductions in ulcer and amputation incidence (22).

On the other hand, VibraTip is a portable, small device used to assess vibration perception on the hallux by delivering a stimulus of 128 Hz. It is mainly used to evaluate large fiber functionality (9). The advantages of the VibraTip are its small size, low cost, and convenience for rapid neurological evaluation in the outpatient context. A study in European patients assessing the diagnostic performance of VibraTip showed moderate to high sensitivity and specificity values (79% and 82%) when compared to vibration perception threshold (VPT) ≥ 25 Volts (V) using a Neurosthesiometer as gold standard for DPSN (23). Another previous study reported a 100% sensitivity and 96.6% specificity of VibraTip compared to VPT ≥ 25 V and the Neuropathy Disability Score (NDS) ≥ 6 (11). The values considered as thresholds in this study were considerably higher than the standard validated diagnostic values and denoted severe DSPN; thus, interpretation of their conclusions is limited. The comparison of our results to the mentioned studies is very difficult since crucial clinical characteristics of patients were not mentioned. Our study showed VibraTip presents a high specificity (91.3%), higher than the study by Bracewell and cols. (23), which may confer this test an important diagnostic power. It also displays a high NVP, which reinforces its potential role as screening test for DSPN. The National Institute for Health and Care Excellence (NICE) evaluated the scientific evidence and cost-effectiveness of VibraTip and considered it is a technology that shows potential to improve the detection of DSPN in diabetes patients, but more research is needed to assess its diagnostic accuracy (24).

To the best of our knowledge, this is the first study to evaluate the diagnostic performance of VibraTip in Latin American patients with T2D. Furthermore, our study provides a complete characterization of T2D patients that may benefit from this tool. Our results suggest VibraTip may be reliable tool to screen for DSPN, and it may even have the diagnostic power to replace instruments as the 128-Hz tuning fork. Its ease to use, portability and ability to produce a consistent vibratory stimulus are advantages that may be beneficial in the outpatient context. We consider that the use of new tools for the early diagnosis of DSPN is necessary in the everyday detection of DSPN, and VibraTip seems an attractive candidate. For the implementation of VibraTip and Neuropad in middle to low-income countries, a crucial factor to take into account is their cost. Compared to other widespread screening tools such as the 10-g monofilament and the tuning fork, VibraTip is cheaper in terms of the cost per device and the estimated cost per-examination (24). Studies assessing the cost-effectiveness of Neuropad as a screening tool for DSPN are lacking; however, its non-reusable nature might implicate larger costs for public health systems.

Our study presents potential limitations that need to be addressed before interpreting our results. First, the sample size is relatively small belonging to a single center; multi-centered, large cohort studies in other Latin American countries are certainly needed to confirm our findings in this population, especially regarding the VibraTip. Second, the patients from our study are elderly (mean age of 70.8 years), and it is possible that the diagnostic performance of the addressed tools changes in younger population. Third, it would be interesting to assess the cost-effectiveness of both VibraTip and Neuropad in middle and low-income countries. Future public health and economic studies addressing this issue are certainly needed.

In conclusion, VibraTip and Neuropad are simple, bedside tests for the screening of DSPN in Latin American T2D patients. VibraTip presents a high diagnostic performance for DSPN, and constitutes a promising candidate for the early diagnosis of this entity in our population.
